# Prevalence of gestational diabetes mellitus according to the different criterias

**DOI:** 10.4274/tjod.38802

**Published:** 2017-03-15

**Authors:** Evren Akgöl, Sedat Abuşoğlu, Faik Deniz Gün, Ali Ünlü

**Affiliations:** 1 Birecik State Hospital, Clinic of Biochemistry, Şanlıurfa, Turkey; 2 Selçuk University Faculty of Medicine, Department of Biochemistry, Konya, Turkey

**Keywords:** Gestational diabetes mellitus, oral glucose tolerance test, diagnostic criteria, prevalence

## Abstract

**Objective:**

The two-step approach recommended by the National Diabetes Data Group (NDDG), Carpenter and Coustan (C&C), and O’Sullivan, and the single-step approach recommended by the International Association of Diabetes and Pregnancy Study Group (IADPSG) are used to diagnose gestational diabetes mellitus (GDM). We aimed to determine GDM prevalence and to compare the two-step and single-step approaches used in the southeastern region of Turkey.

**Materials and Methods:**

In total, 3048 records of pregnant women screened for GDM between 2008 and 2014 were retrospectively extracted from our laboratory information system. GDM was defined according to the criteria of NDDG, C&C, and O’Sullivan between in 2008 and 2011, and according to those of the IADPSG between 2012 and 2014. Demographic variables were compared using student’s t-test. The linear trends in GDM prevalence with age were calculated using logistic regression.

**Results:**

GDM prevalence was found as 4.8%, 8%, and 13.4% using the NDDG, C&C, and O’Sullivan two-step approach, respectively, and 22.3% with the IADPSG single-step approach. GDM prevalence increased with increasing age in both approaches.

**Conclusion:**

GDM prevalence was higher using the single-step approach than with the two-step approach. There was a significant increase in GDM prevalence using the IADPSG criteria.

## INTRODUCTION

Gestational diabetes mellitus (GDM) is one of the most common medical complications of pregnancy. GDM is defined as glucose intolerance with onset or first recognition during pregnancy and is a well-established risk factor for adverse infant health outcomes, including fetal macrosomia, birth trauma, neonatal hypoglycemia, and fetal death^(1,2)^.

The initial criteria for GDM were established by O’Sullivan and Mahan in 1964. In this criteria, two or more abnormal glucose values in the 3-h, 100-g oral glucose tolerance test (OGTT) were considered pathological^(3)^. In 1979 and 1982, the National Diabetes Data Group (NDDG) and Carpenter and Coustan (C&C), respectively, recommended new diagnostic thresholds for the 100-g OGTT. These approaches are still used for pregnant women who have a high glucose challenge test (GCT) result^(4,5)^. More recently, after an extensive analyses of the Hyperglycemia and Adverse Pregnancy Outcomes study, the International Association of Diabetes and Pregnancy Study Group (IADPSG) recommended a single-step approach and new diagnostic criteria for GDM that was based on a 2-h, 75-g OGTT^(6,7)^. However, in general practice, this approach is still controversial. The American Diabetes Association and World Health Organization have adopted the IADPSG recommendation, whereas the American College of Obstetricians and Gynecologists advises continuing with the two-step screening strategy^(8,9,10)^.

GDM prevalence varies widely depending on the population studied, age, and the diagnostic test employed. In Turkey, the prevalence ranges from 1.2% to 4.48% according to the criteria of NDDG and C&C. However, there are no data on GDM prevalence using the new, single-step approach.

Our aim was to determine GDM prevalence and to compare the two-step approach with the single-step approach among a population from the southeastern region of Turkey.

## MATERIALS AND METHODS

This study was approved by the Ethics Committee of Selçuk University Faculty of Medicine on September 8^th^, 2015 (approval number: 2015/267).

This retrospective study was conducted between January 2008 and December 2014 in the Birecik State Hospital, Şanlıurfa, which is located in the southeastern region of Turkey and provides service to approximately 150000 people. All women who were non-diabetic, between 24 and 28 weeks’ pregnancy, and aged between 15 and 49 years were screened for GDM using a two-step approach between January 2008 and December 2011, and the single-step approach between January 2012 and December 2014. During the two-step approach, all pregnant women were screened for GDM with the 50-g, 1-h GCT. A positive GCT result was defined as a serum glucose level of ≥140 mg/dL. Patients with a positive GCT underwent a 3-h, 100-g diagnostic OGTT. Patients with two or more elevated glucose results from the diagnostic OGTT were diagnosed as having GDM according to the criteria of O’Sullivan [i.e. fasting plasma glucose (FPG) level: ≥90 mg/dL, 1 h: ≥165 mg/dL, 2 h: ≥145 mg/dL, and 3 h: ≥125 mg/dL], NDDG (FPG: ≥105 mg/dL, 1 h: ≥190 mg/dL, 2 h: ≥165 mg/dL, and 3 h: ≥145 mg/dL), and C&C (FPG: ≥95 mg/dL, 1 h: ≥180 mg/dL, 2 h: ≥155 mg/dL, and 3 h: ≥140 mg/dL). In the single step approach, patients were screened for GDM with a 2-h, 75-g OGTT. GDM was diagnosed by a single elevated 2-h, 75-g glucose tolerance test (FPG: ≥92 mg/dL, 1 h: ≥180 mg/dL and 2 h: ≥153 mg/dL) as defined by the IADPSG.

### Statistical Analysis

The records of pregnant women screened for GDM were extracted from the laboratory information system. All glucose measurements in patient samples were performed using the hexokinase method. Demographic variables were compared using student’s t-test. Linear trends with age and GDM prevalence were calculated using logistic regression. Statistical analyses were performed using SPSS v16. A p-value <0.05 was considered significant.

## RESULTS

A total of 1385 pregnant women were screened for GDM with a two-step approach between January 2008 and December 2011. Of these women, 501 (36.2%) were found at risk for GDM during GCT and were included in the 3-h, 100-g diagnostic OGTT. During the diagnostic OGTT, 66 of the 501 patients were diagnosed as having GDM according to the criteria of NDDG and 111 and 185 of 501 patients were diagnosed as having GDM according to the criteria of C&C and O’Sullivan, respectively. GDM prevalence was found as 4.8%, 8%, and 13.4% based on the criteria of NDDG, C&C, and O’Sullivan, respectively, during the two-step approach. GDM prevalence for each year is presented in Table 1. A total of 1663 pregnant women were screened for GDM using the single-step approach between January 2012 and December 2014, and 371 were diagnosed as having GDM. The GDM prevalence rate was found as 22.3% according to the criteria of IADPSG. The prevalence rates for each year are presented in Table 2.

In our study, patients who were diagnosed as having GDM were significantly older than healthy patients (Table 3). GDM prevalence increased with increasing age with both approaches (Table 4).

## DISCUSSION

GDM prevalence may differ depending on the population being screened and the diagnostic test being performed. GDM prevalence was reported as 8.8% using the NDDG criteria and 10.6% using the C&C criteria in Spain^(11,12)^. GDM prevalence was also determined as 5.5% in the United States of America (USA), 8.4% in China, and 7.7% in Morocco according to the C&C criteria^(13,14,15)^.

In studies conducted in different regions of Turkey, GDM prevalence was found between 1.23% and 4.2% according to the criteria of the NDDG, and between 2% and 4.48% according to the C&C criteria^(16,17,18,19,20)^. In our study, GDM prevalence was found as 4.8% and 8% using the NDDG and C&C criteria, respectively, which is higher than that those reported in previous Turkish studies. The higher GDM prevalence is probably due to regional dietary habits. GDM was higher using the criteria of C&C than with the NDDG criteria. This increase may result from the increased sensitivity of the test when using the C&C criteria because its glucose value for a diagnosis of GDM is lower than in the NDDG criteria.

After IADPSG issued a consensus statement on the new criteria for the diagnosis of GDM, GDM prevalence significantly increased when the new criteria were adopted^(12,21,22)^. GDM prevalence increased 3.3 times in Spain (10.6% to 35.5%), 2.8 times in the USA (5.5% to 15.6%) and 2.25 times in China (8.4% to 18.9%) using the new IADPSG criteria^(12,13,14)^.

In Turkey, our study is the first to determine GDM prevalence using the new, single-step approach. Similar to other studies, we found that GDM prevalence using the IADPSG single-step approach increased the positivity rate as much as 4.5 times than that of the two-step NDDG approach, 3 times more than the two-step C&C approach, and 1.7 times more than the two-step O’Sullivan approach. This significant increase results from the fact that only one elevated result is sufficient to make the diagnosis, not two. In our study, the increase in GDM prevalence with IADPSG was 2 times higher in a subgroup of women aged <30 years.

In our study, GDM prevalence increased significantly with increasing age, regardless of the criteria used. A similar association has been observed in various studies^(16,23,24)^. GDM prevalence in women aged >30 years was 4.2 times greater than that of women aged ≤30 years using the C&C and NDDG criteria, 2.5 times greater using the criteria of O’Sullivan, and 2 times greater using the IADPSG criteria. This means that the criteria of O’Sullivan and IADPSG diagnose more younger women (i.e. women <30 years) as having GDM.

## CONCLUSION

Therefore, the new IADPSG criteria provide a higher GDM prevalence and diagnose more young women. This may also be effective in decreasing the medical disbursement for the treatment of the disease; however, the benefits of these findings are still unclear. New prospective studies may highlight the outcomes of new approaches by the criteria of IADPSG.

## Figures and Tables

**Table 1:**
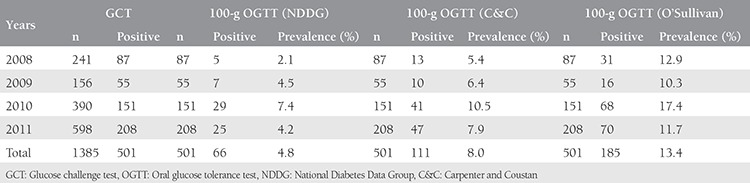
Gestational diabetes mellitus prevalence according to the two-step approaches by year

**Table 2:**
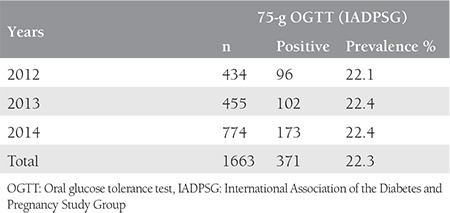
Gestational diabetes mellitus prevalence according to the single-step approach by year

**Table 3:**
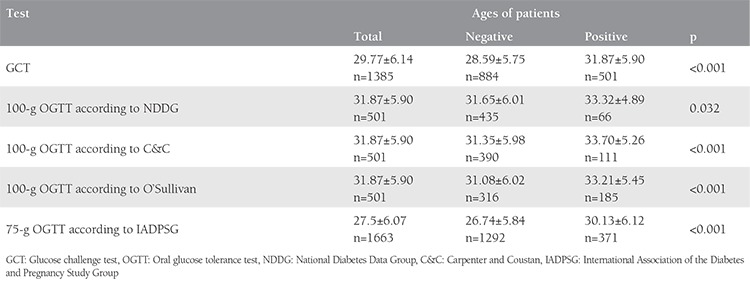
Age characteristics of healthy patients and patients with gestational diabetes mellitus

**Table 4:**
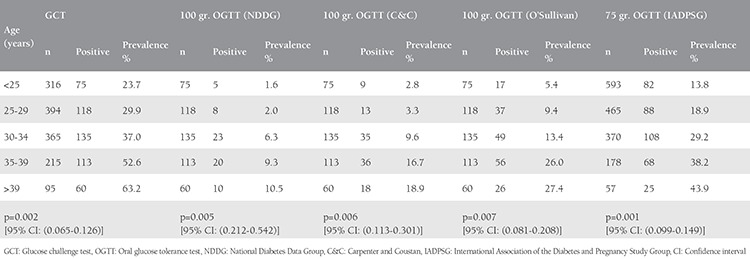
The trends in the prevalence of gestational diabetes mellitus with age

## References

[1] no author (1997). Report of the Expert Committee on the Diagnosis and Classification of Diabetes Mellitus. Diabetes Care.

[2] Galarneau F, Inzucchi SE (2004). Diabetes mellitus in pregnancy. Obstet Gynecol Clin North Am.

[3] O’Sullivan JB, Mahan C (1964). Criteria for oral glucose tolerance test in pregnancy. Diabetes.

[4] no author (1979). Classification and diagnosis of diabetes mellitus and other categories of glucose intolerance. Diabetes.

[5] Carpenter MW, Coustan DR (1982). Criteria for screening tests for gestational diabetes. Am J Obstet Gynecol.

[6] Metzger BE, Lowe LP, Dyer AR, Trimble ER, Chaovarindr U, Coustan DR, et al (2008). Hyperglycemia and adverse pregnancy outcomes. N Engl J Med.

[7] Metzger BE, Gabbe SG, Persson B, Buchanan TA, Catalano PA, Damm P, et al (2010). International Association of Diabetes and Pregnancy Study Groups Consensus Panel. International association of diabetes and pregnancy study groups recommendations on the diagnosis and classification of hyperglycemia in pregnancy. Diabetes Care.

[8] American Diabetes Association (2011). Executive summary: standards of medical care in diabetes 2011. Diabetes Care.

[9] World Health Organization (2013). Guidelines Approved by the Guidelines Review Committee.

[10] no author (2011). Committee on Obstetric Practice, Screening and diagnosis of gestational diabetes mellitus. Obstet Gynecol.

[11] Ricart W, Lopez J, Mozas J, Pericot A, Sancho MA, Gonzalez N, et al (2005). Potential impact of American Diabetes Association (2000) criteria for diagnosis of gestational diabetes mellitus in Spain. Diabetologia.

[12] Duran A, Saenz S, Torrejon MJ, Bordiu E, Del Valle L, Galindo M, et al (2014). Introduction of IADPSG Criteria for the Screening and Diagnosis of Gestational Diabetes Mellitus Results in Improved Pregnancy Outcomes at a Lower Cost in a Large Cohort of Pregnant Women: The St. Carlos Gestational Diabetes Study. Diabetes Care.

[13] Ogunleye O, Davidson K, Gregg A, Egerman R (2014). Two-Step Glucose Tolerance Test Compared With One-Step Glucose Tolerance Test. Obstet Gynecol.

[14] Yumei W, Huixia Y, Weiwei Z, Hongyun Y, Haixia L, Jie Y, et al (2014). International Association of Diabetes and Pregnancy Study Group criteria is suitable for gestational diabetes mellitus diagnosis: further evidence from China. Chin Med J (Engl).

[15] Macaulay S, Dunger DB, Norris SA (2014). Gestational Diabetes Mellitus in Africa: A Systematic Review. PLoS One.

[16] Karcaaltincaba D, Kandemir O, Yalvac S, Güvendag-Güven S, Haberal A (2009). Prevalence of gestational diabetes mellitus and gestational impaired glucose tolerance in pregnant women evaluated by National Diabetes Data Group and Carpenter and Coustan criteria. Int J Gynaecol Obstet.

[17] Tanir HM, Sener T, Gürer H, Kaya M (2005). A ten-year gestational diabetes mellitus cohort at a university clinic of the mid-Anatolian region of Turkey. Clin Exp Obstet Gynecol.

[18] Turgut A, Ünsal Boran S, Dolgun ZN, Acıoğlu H, Yaman Görük N (2011). The frequency of gestational diabetes mellitus in a maternity hospital antepartum clinic. Dicle Medical Journal.

[19] Erem C, Cihanyurdu N, Deger O, Karahan C, Çan G, Telatar M (2003). Screening for gestational diabetes mellitus in northeastern Turkey (Trabzon City). Eur J Epidemiol.

[20] Aydın M, Gürel A, Celik C, Tülübas F, Abalı R, Yılmaz A (2013). The prevalence of gestational diabetes mellitus in Namik Kemal University Training and Research Hospital. Yeni Tıp Dergisi.

[21] Benhalima K, Hanssens M, Devlieger R, Verhaeghe J, Mathieu C (2013). Analysis of pregnancy outcomes using the new IADPSG recommendation compared with the Carpenter and Coustan criteria in an area with a low prevalence of gestational diabetes. Int J Endocrinol.

[22] Moses RG, Morris GJ, Petocz P, San Gil F, Garg D (2011). The impact of potential new diagnostic criteria on the prevalence of gestational diabetes mellitus in Australia. Med J Aust.

[23] Guariguata L, Linnenkamp U, Beagley J, Whiting DR, Cho NH (2014). Global estimates of the prevalence of hyperglycaemia in pregnancy. Diabetes Res Clin Pract.

[24] Ferrera A (2007). Increasing prevalence of gestational diabetes mellitus: a public health perspective. Diabetes Care.

